# A preliminary study on calcifying nanoparticles in dental plaque: Isolation, characterization, and potential mineralization mechanism

**DOI:** 10.1002/cre2.885

**Published:** 2024-05-26

**Authors:** Siwei Wang, Lan Yang, Guohui Bai, Yu Gu, Qin Fan, Xiaoyan Guan, Jie Yuan, Jianguo Liu

**Affiliations:** ^1^ Department of Dental Implantology, The Affiliated Stomatological Hospital Zunyi Medical University Zunyi China; ^2^ Department of Prosthodontics, The Affiliated Stomatological Hospital Zunyi Medical University Zunyi China; ^3^ Key Laboratory of Oral Disease of Higher Schools in Guizhou Province Zunyi Medical University Zunyi China; ^4^ Department of Stomatology Zunyi Medical University Zhuhai China; ^5^ Department of Orthodontics, The Affiliated Stomatological Hospital Zunyi Medical University Zunyi China; ^6^ Department of Pain Medicine The Affiliated Hospital of Zunyi Medical University Zunyi China

**Keywords:** calcifying nanoparticles, dental plaque, nanobacteria, periodontal disease

## Abstract

**Objectives:**

Calcifying nanoparticles (CNPs), referred to as nanobacteria (NB), are recognized to be associated with ectopic calcification. This study aims to isolate and culture CNPs from the dental plaque of patients with periodontal disease and investigate their possible role in unravelling the aetiology of periodontal disease.

**Material and Methods:**

Supragingival and subgingival plaques were sampled from 30 periodontitis patients for CNPs isolation and culture. Alkaline phosphatase (ALP) content changes were tracked over time. Positive samples underwent thorough morphological identification via hematoxylin and eosin (HE) staining, Alizarin red S (ARS), and transmission electron microscopy (TEM). The chemical composition of CNPs analysis involved calcium (Ca) and phosphorus (P) content determination, Fourier transform infrared spectroscopy (FTIR), and X‐ray diffraction (XRD).

**Results:**

The subgingival plaque dental group exhibited a higher CNPs isolation rate at 36.67% (11/30) compared to the supragingival dental plaque group at 66.67% (20/30). ALP activity varied among the positive, negative and control groups. Morphological observation characterized the CNPs as round, oval, and ellipsoid particles with Ca deposits. Chemical analysis revealed the Ca/P ratio was 0.6753. Hydroxyl, methyl, carbonate, phosphate, hydrogen phosphate, and dihydrogen phosphate were detected by FTIR; the main chemical components detected by XRD were hydroxyapatite and tricalcium phosphate.

**Conclusion:**

CNPs were found in periodontitis‐related dental plaque and exhibited the potential to develop calcified structures resembling dental calculus. However, the potential involvement of ALP in CNPs formation requires deeper exploration, as does the precise nature of its role and the interrelation with periodontitis demand a further comprehensive investigation.

## 1 INTRODUCTION

“Nanobacteria” (NB) refers to an ultrastructural bacterium measuring 20–500 nm in diameter, the smallest known replicating entities of organic life on earth discovered by the Kajander group Çiftçioglu & Kajander, 1998; Ciftcioglu et al., 1997). It emerged as a potential pathogen associated with an array of conditions including kidney stones, prostatitis, atherosclerotic plaques etc. Shen et al. (2010); Puskás et al. (2005) Notably, NB is covered with hydroxyl apatite, passes through 0.1‐mm filters and eludes detection through conventional microbiological methods. Its classification within the α‐2 subgroup of proteobacteria, a group also encompassing Bartonella and Brucella, is attributed to the presence of the 16 S rRNA gene sequences Kajander & Iftcioglu, 1998). Initial investigations underscored NB's distinctive role as a nucleation site for the formation of biogenic apatite structures. Moreover, it releases toxins and cytokines that prompt cellular damage and trigger inflammatory responses in tissues (Hjelle et al., 2000; Kajander et al., 2003; Zhang et al., 2013).

However, as research advances, an alternative perspective has emerged, considering that NB might be a nonliving mineral‐protein particle exhibiting structure, growth, proliferation, and subculture qualities similar to living microorganisms (Cisar et al., 2000; Martel et al., 2014; Schlieper et al., 2011; Wong et al., 2015). Multiple research teams have undertaken experimental endeavours, highlighting that the monoclonal antibodies targeting NB share cross‐antigens with albumin and fetuin‐A and broad‐range polymerase chain reaction amplification has failed to detect bacterial DNA. In vitro, CaCO_3_ precipitates can replicate NB‐like nano‐calcifying nanoparticles. Furthermore, a dual inhibitory‐seeding mechanism involving proteinaceous factors was proposed (Martel & Young, 2008; Wu et al., 2009; Young et al., 2009). Therefore, given the absence of robust nucleic acid sequences, scholars advocate for designating NB as calcifying nanoparticles (CNPs) (Kajander, 2006). Over the past decade, the comprehension of CNPs has expanded. Following, the role of CNPs within the gut lumen has been elucidated through mice experimental models, the complex granules have been detected in the calcified arteries of diabetic patients and calciprotein particles have been implicated in promoting cytotoxicity in renal epithelial cells (Kunishige et al., 2020; Powell et al., 2015; Wu et al., 2020). Despite the controversies, CNPs potentially signify precursors to calcification within the human body, thus providing significant insights into the mechanism of pathological calcification (Martel et al., 2018).

Periodontal disease, a major inflammatory disease of the oral mucosa, causes severe loss of supporting structures and substantial tooth loss, affecting 10–15% of the global population (Kinane et al., 2017). The triggering factor leading to periodontal tissue inflammation and destruction is dental plaque—a microbial colony on the tooth surface, primarily manifesting an embedded‐type biofilm comprising bacteria and a polymer matrix with a complex ecological environment. The subsequent mineralization of the dental plaque results in the formation of calculus which is one of the important local contributing aspects to periodontal disease (Jakubovics et al., 2021; Akcalı & Lang, 2018). Concurrently, it's imperative to acknowledge that periodontitis is intricately connected with various chronic systemic disorders, encompassing cardiovascular disease, type 2 diabetes mellitus (T2DM), and rheumatoid arthritis, among others (Hajishengallis & Chavakis, 2021). Hence, the revelation of CNPs initiates novel avenues for investigating the causes of dental calculus formation and fresh pathways for understanding the complex interactions between dental plaque and dental calculus. This development not only fosters a deeper comprehension of the molecular mechanisms behind periodontitis but may also serve as a conduit to reveal the intricate links linking periodontal illnesses with systemic diseases. Thus far, CNPs or NB‐like particles have been isolated from dental pulp stones, gingival crevicular fluids and saliva. Besides, they have been reported to be internalized by both gingival epithelial cells and monocyte‐derived macrophages (Peng et al., 2021; Sharma, 2013; Yang et al., 2011; Zhang et al., 2009).

**Figure 1:**
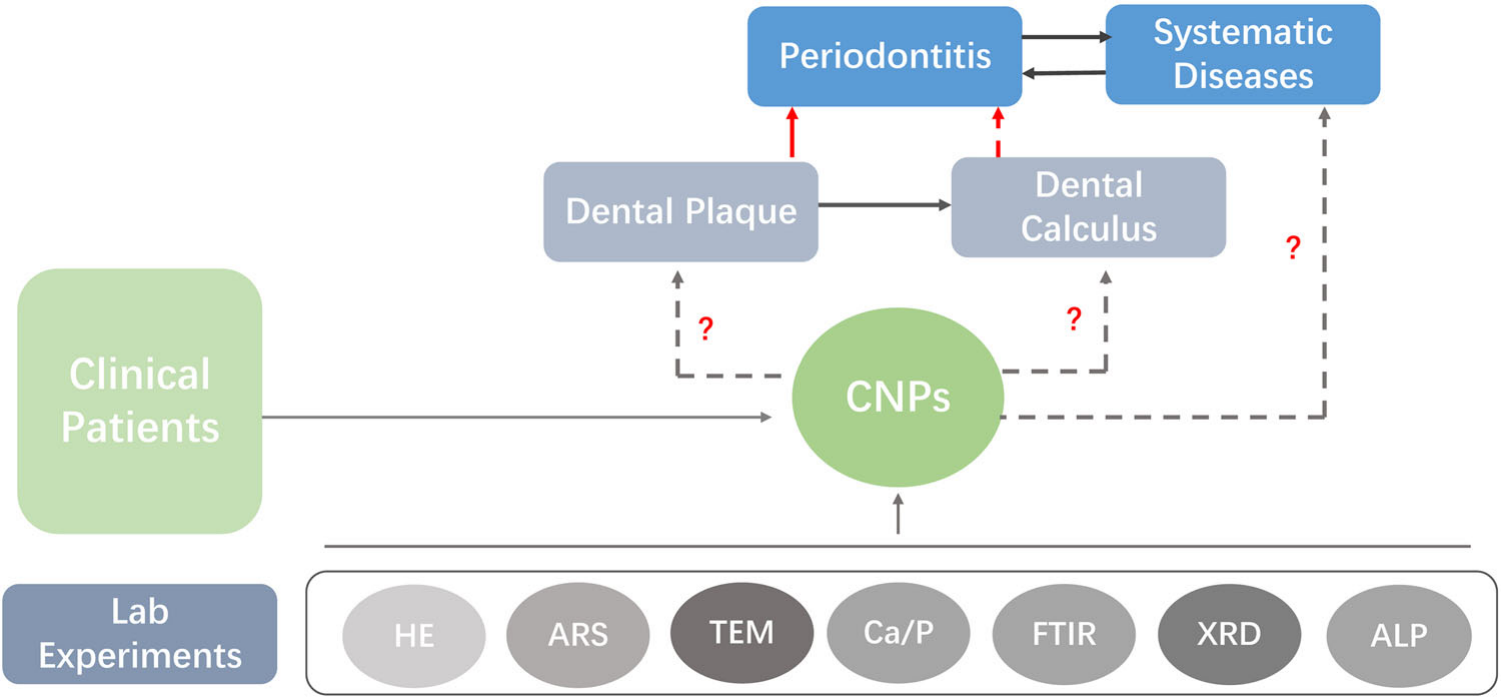
The Experimental Flowchart. Different experimental techniques were used to reveal the correlation between CNPs, periodontal disease and systematic diseases.

In this study, an attempt was made to isolate and culture CNPs from dental plaque of periodontitis patients for morphological observation and chemical composition analysis and to monitor the changes in alkaline phosphatase (ALP) content, so as to investigate the possible mineralization mechanism of CNPs (Figure 1). These findings may provide a theoretical basis for further investigation of the correlation between CNPs and periodontal disease.

## 2 MATERIAL AND METHODS

### 2.1 Dental plaque collection

Supragingival and subgingival dental plaque samples were individually obtained from a cohort of thirty patients, aged between 37 and 61 years (comprising 17 males and 13 females, with a mean age of 50.07 ± 6.56 years). These patients were diagnosed with chronic periodontitis in accordance with international diagnostic criteria (Tonetti et al., 2018). This study was conducted according to the Declaration of Helsinki and the participants were drawn from the Department of Periodontics over a period spanning 4 months. Before their involvement, comprehensive information regarding the study was provided to each prospective participant, and informed consent was acquired through the completion of a signed agreement. All participants were of legal age, exceeding 18 years. For every subject, supragingival and subgingival dental plaque samples were collected from an average of three tooth positions based on the results of the probing. Each sample was placed in a 1.5 ml microtube with 0.5 ml sodium mercaptoethanol pre‐reduction transfer solution and was transported back to the laboratory in a dry ice box.

### 2.2 Culture of CNPs from dental plaque

The samples were placed in a vortex shaker for the 20 s to evenly disperse the plaque mass, followed by dilution with a tenfold volume of sodium mercaptoethanol (Sangon, CA), filtration with a 0.22‐μm filter (Gibco, US), and centrifugation at 14,000 g, 4°C for 20 min (Tremo, US). After centrifugation, the supernatant was discarded, and the bottom pellet was rinsed with phosphate‐buffered saline (PBS) three times, followed by centrifugation for 10 min each time. Finally, the solution containing isolated CNPs was obtained after 1 ml of the liquid and the pellet was retained and filtered with a 0.22‐μm filter. The filtrate was cultured in a flask containing two volumes of modified 1640 medium containing (Hyclone, US) 10% fetal bovine serum (FBS; Sigma) as described in previous studies (Çiftçioglu & Kajander, 1998). In the control group, sterile PBS was used instead of the isolated CNP solution, and the other components were the same. All prepared samples were followed by culturing in a 37°C, 5% CO2 cell incubator (Thermo, US).

### 2.3 The dynamic changes and determination of ALP activity of CNPs

The cultures were incubated for 12 weeks. The optical density (OD) values were recorded using a spectrophotometer (TU‐1901, CA) at a wavelength of 650 nm and the ALP content was measured in the supernatants of all samples and the controls at weeks 1, 2, 3, 4, 6, 8, and 12 of culture. The presence of macroscopic white precipitates was considered culture‐positive. Hence, the positive and negative samples of supragingival dental plaque (SUPDP) were classified as A1 and A2, while those in subgingival dental plaque (SUBDP) were marked as groups B1 and B2. The control group was set as group C.

### 2.4 Morphological observation of CNPs

The positive samples were macroscopically observed, followed by centrifugation at 14,000 g and 4°C for 20 min, washed with PBS, natural drying, fixation and photographed under a microscope (Olympus, Tokyo). For hematoxylin and eosin (HE) staining, hematoxylin staining was performed for 2–3 min, the slides were rinsed in tap water, and eosin staining was performed for 1 min. The slides were then rinsed and dried followed by neutral gum mounting. Alizarin Red S (ARS) staining (GenMed, US) involved 75% ethanol fixation for 10 min, rinsing with distilled water, incubation in alizarin red staining solution at 37°C for 10 min, drying, and neutral gum mounting. In the context of transmission electron microscopy (TEM), 2.5% glutaraldehyde was added to the pellet for fixation for 2 h, fixed with 1% osmic acid for 1 h, rinsed, and the slide was stained followed by gradient dehydration. With an acetone/embedding solution ratio of 1:4, the samples were placed into the oven at 37°C overnight, followed by embedding and polymerization at 45°C for 3 h, photographs were taken using TEM (Hitachi, Tokyo) for morphological observation.

### 2.5 Chemical composition analysis of CNPs

Ten CNP culture‐positive samples were centrifuged at 14,000 g and 4°C for 20 min, the supernatant was discarded, and the samples were washed three times with sterile PBS for 5 min each time. Then, the supernatant was discarded, and 0.1 g of the pellet was measured for flame atomic absorption and ammonium molybdate spectrophotometer assays (Varian, US), respectively. Fourier‐transform infrared spectroscopy (FTIR, Varian, US) was used to detect CNP‐related functional groups. X‐ray diffraction (XRD, Rigaku, Tokyo) was used to analyze the crystal morphology and crystal phase composition of CNPs.

### 2.6 Statistical analysis

Statistical software SPSS 29.0 (IBM Corp., Armonk, NY) was used for the analyses. Continuous variables are represented by the mean standard deviation (SD), and categorical variables are represented by percentages. The two‐independent sample test, the Chi‐Square test (2 test), one‐way ANOVA, and Student‐Newman‐Keuls (SNK) were used to compare the variables. 0.05 probability (p) values were considered significant.

## 3 RESULTS

### 3.1 The isolation and culture results of CNPs

**Figure 2:**
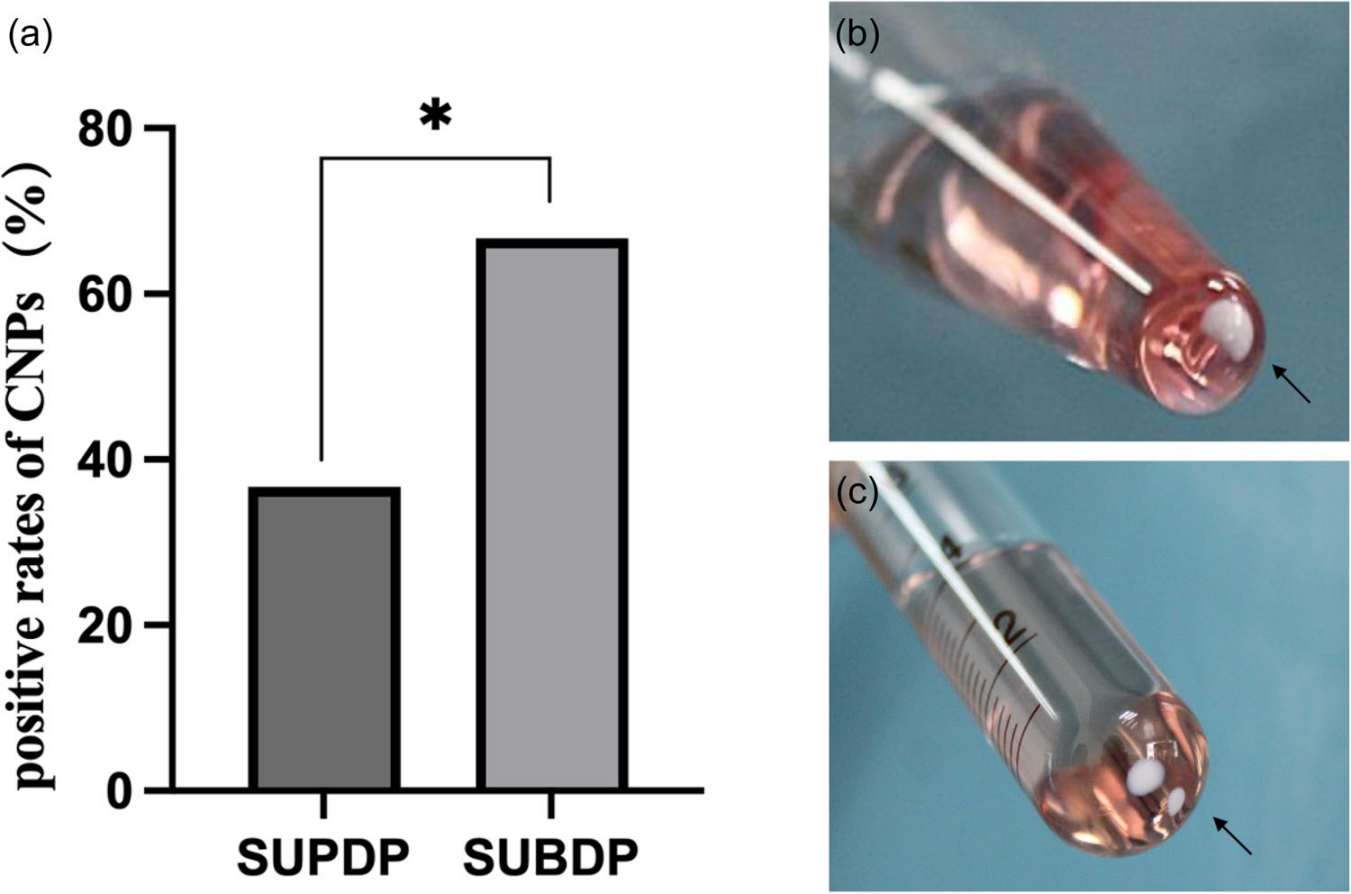
Isolation rate and Cultivation types of CNPs. (a) Comparison of CNP positive rates in the supragingival and subgingival plaques. The isolation rates of CNPs were 36.67% (11/30) in the SUPDP group and 66.67% (20/30) in the SUBDP group, *comparison with the SUPDP group（*p*＜0.05); (b) The white precipitates were harvested and a single clump was clustered at the bottom; (c) several clumps formed were clustered at the bottom.

During incubation, the isolation rates of CNPs were 36.67% (11/30) in the SUPDP group and 66.67% (20/30) in the SUBDP group, and the difference between the two groups was statistically significant (χ2 = 4.27, *p* < .05) (Figure 2a). Besides, the observed spontaneous crystallization (Figure 2b, c) produced by the culture‐positive samples was classified into four types: (i) single clump was clustered at the bottom of the test tube and closely clung to the walls; (ii) clumps were clustered at the bottom of the test tube and were partially suspended upon light shaking; (iii) clumps were clustered at the bottom of the test tube and completely suspended upon light shaking; and (iv) several clumps formed were clustered at the bottom of the test tube and closely clung to the walls. No precipitate was produced in the control group.

**Figure 3:**
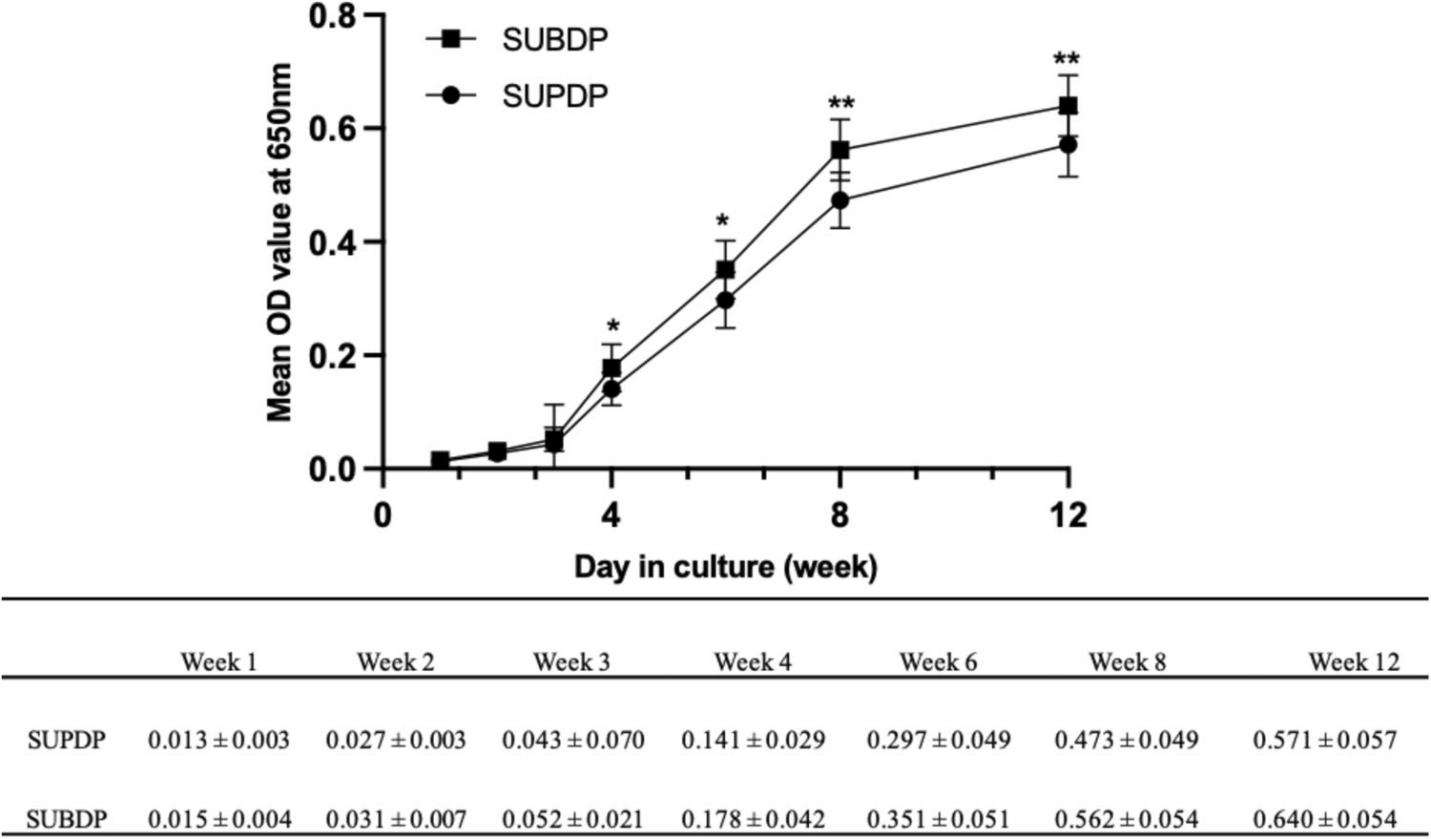
The growth curves of CNPs. *Comparison with SUPDP group (*p* < .05), **Comparison with SUPDP group (*p* < .01).

The growth curves of CNPs in the two groups were then plotted with the mean OD of culture‐positive samples as the ordinate and the culture time (weeks) as the abscissa (Figure 3). The concentration of CNPs increased over time, without a significant decay trend. There was a statistical difference in the OD values of growth density at weeks 4 and 6 of culture (*p* < .05) between the two groups and this difference was more significant at 8 and 12 weeks (*p* < .01).

### 3.2 ALP activity assay

**Figure 4:**
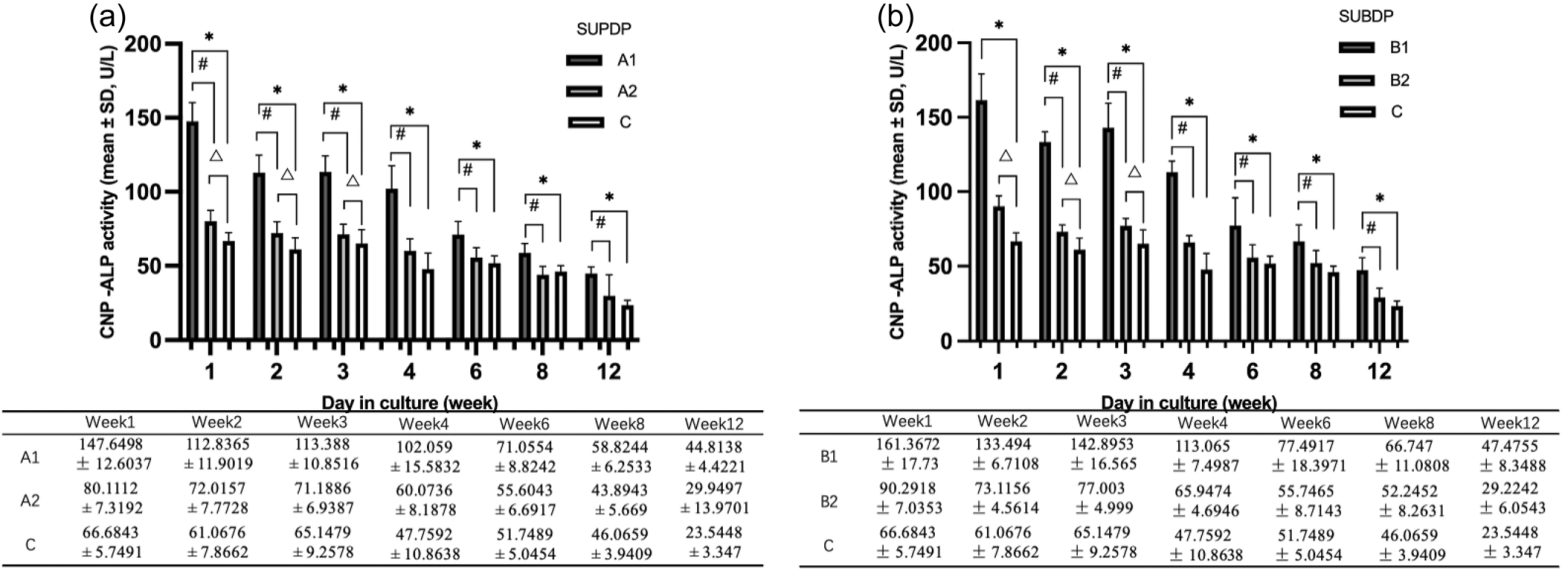
Determination of CNPs‐ALP activity at different time points. (a) *There was a statistically difference between groups A1 and C, *p* < .01; # A significantly higher ALP level was found in group A1 compared to group A2, *p* < .01; △Comparison between group A2 and group C, *p* < .01; (b) *There was a statistical difference between groups B1 and C, *p* < .01: # A significantly higher ALP level was found in group B1 compared to group B2, *p* < .01: △Comparison between groups B2 and C, *p* < .01.

In the SUPDP group, the ALP activity in A1 was higher than that in A2 and C from weeks 1 to 12 of culture (*p* < .01). Besides, A2 was higher than C in weeks 1‐3(*p* < .01) (Figure 4a). In the context of the SUBDP group, the ALP activity in B1 was pronounced differently compared to C in the culture period (*p* < .01). In addition, there were significant differences between B1 and B2 from 2 to 12 weeks (*p* < .01). Moreover, the statistical difference was between B2 and C in the initial 3‐week period (Figure 4b).

### 3.3 Light microscopy, HE staining, ARS staining and TEM results of CNPs

**Figure 5:**
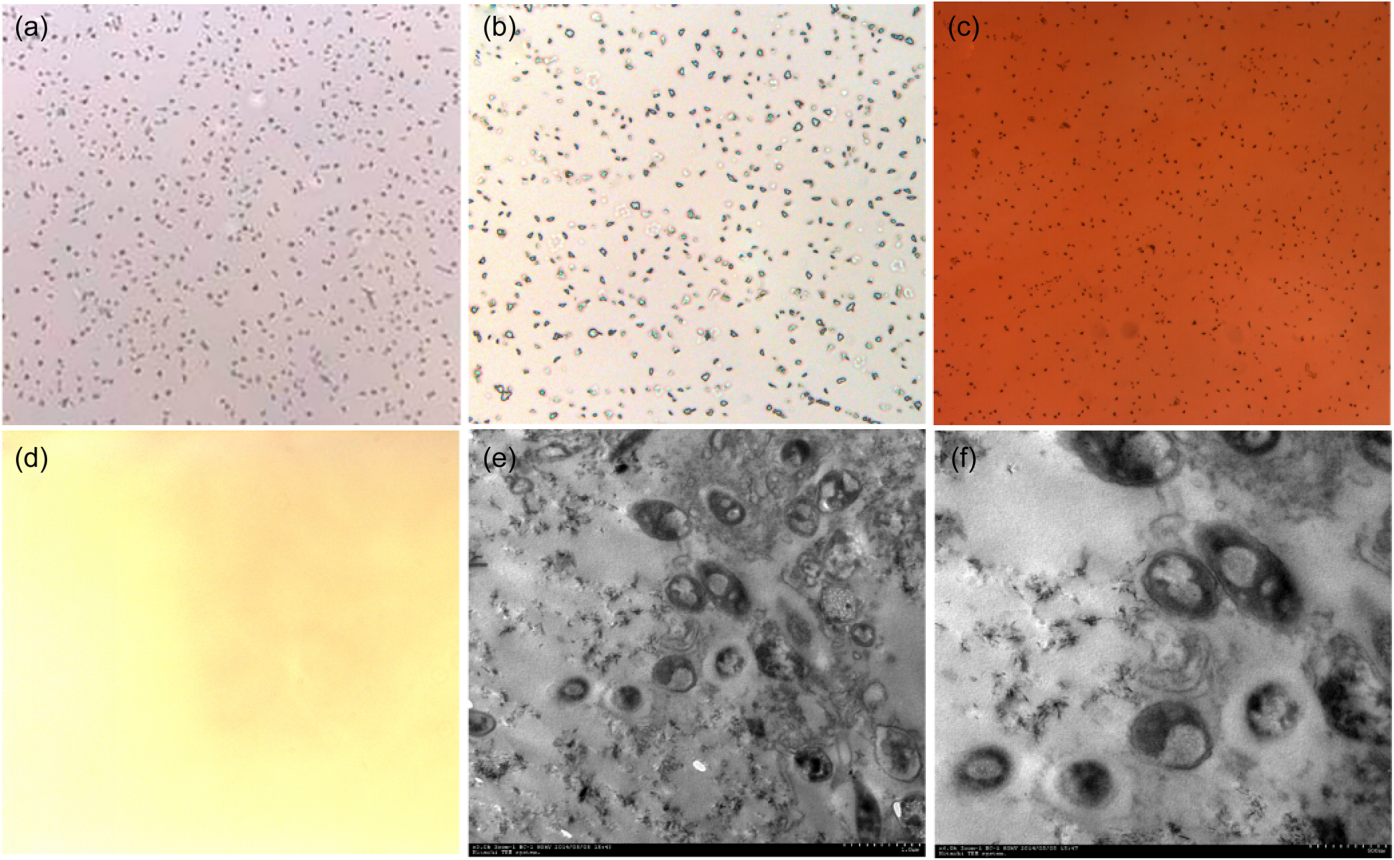
The morphological identification of dental plaque CNPs culture from patients with periodontal diseases. (a) Light microscopy (×200); (b) HE staining (×200); (c) ARS staining (×200); (d) PBS control (×200); (e) TEM 1.0 μm; (f) TEM 0.5 μm.

CNPs appeared as clustered small particles under a light microscope (Figure 5a). HE staining showed dark blue staining around the small particles (Figure 5b), ARS staining showed dark orange round and oval small particles (Figure 5c), and no granular material was seen in the PBS control group (Figure 5d). Under TEM, the CNP cultures were round, oval, and ellipsoid particles. Vacuolization was observed in the centre of the particles, which were surrounded by a high‐density linear shadow, and there were fine scattered secretions between the particles (Figure 5e, f).

### 3.4 Determination of ca and P in CNPs

The contents of Ca and P in 10 culture‐positive CNPs were determined by a flame atomic absorption spectrometer and ammonium molybdate spectrophotometer. The Ca concentration and content were 1.2909 ± 0.4340 mmol/L and 7.7454 ± 2.604 nmol/mg, and the P concentration and content were 1.9116 ± 2.5630 mmol/L and 11.4696 ± 3.9084 nmol/mg, respectively. The Ca/P ratio was 0.6753 (Table 1).

**Table 1:** CNP determination of Ca and P content.

Element	Number	Line equation	Correlation index	Concentration (mmol/L)	Content (nmol/mg)
Ca	10	Y = 0.0146x + 0.0037	0.9986	1.2909 ± 0.4340	7.7454 ± 2.6040
P	10	Y = 0.0187x + 0.0023	0.9991	1.9116 ± 2.5630	11.4696 ± 3.9084

### 3.5 FTIR detection of CNP‐related groups

**Figure 6:**
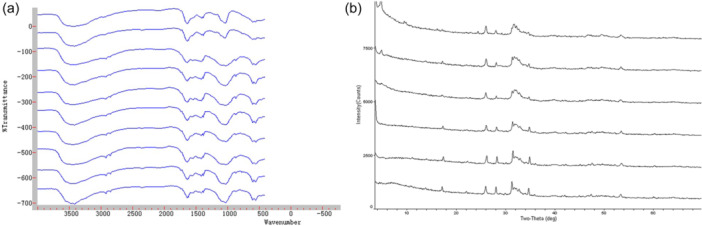
The chemical composition analysis of CNPs with FTIR and XRD. (a) FTIR detection of CNP‐related groups; (b) XRD detection of the crystal habit of CNPs.

Related functional groups were detected by FTIR in 10 CNP‐positive samples. The stretching vibrations in the spectra were mainly in the absorption bands of 3670‐3230 cm‐1 (s), 2975‐2845 cm‐1 (s), 1450‐1410 cm‐1 (s), and 1100‐1000 cm‐1 (s), corresponding to the following groups: hydroxyl (‐OH), methyl (‐CH_3_), carbonate (CO_3_^2^‐), phosphate (PO_4_^3^‐), hydrogen phosphate (HPO_4_‐), and dihydrogen phosphate (HPO_4_^2^‐) (Figure 6a).

### 3.6 Crystal morphology of CNPs as measured by XRD

Six CNP‐positive samples were detected by XRD, and their main chemical components were hydroxyapatite (HA, Ca8.86(PO_4_)_6_(H_2_O)_2_) and tricalcium phosphate (Ca_3_(PO_4_)_2_)_2_) as determined by database comparison (Figure 6b).

## 4 DISCUSSION

### 4.1 CNPs isolation and cultivation

In the present study, we successfully isolated CNPs‐like entities from both supragingival and subgingival dental plaque samples originating from patients afflicted with periodontal disease. Notably, a greater abundance of CNPs was observed within the subgingival plaque, thereby prompting an exploration into the potential association between CNPs and periodontitis. Plausible explanations for this phenomenon may exist in the content of inorganic components in plaque (Shokeen et al., 2022), including calcium and phosphate ions, which could provide the growth impetus for CNPs formation and advantage to initiate self‐replication and form an HA shell process. Besides, the alkaline products metabolized by nitrogen compounds are important factors in regulating the pH of dental plaque (Jin & Yip, 2002). Processes such as the urea metabolic pathway and arginine decomposition pathway contribute to pH modulation within the plaque, thereby engendering elevated concentrations of inorganic ions such as calcium and phosphorus, consequently fostering a favourable environment for CNP proliferation (D'souza et al., 2023; Wong et al., 2002). Moreover, it is crucial to consider the intricacies of the microecological habitat within the subgingival plaque, characterized by a predominantly anaerobic environment often localized within the gingival pocket (Abiko et al., 2010). The oxidation–reduction potential was low, which made cleaning difficult, and such an environment may be more conducive to CNP colonization (Ding et al., 2023).

In terms of precipitation morphology, four aggregation forms of CNPs in dental plaque were observed from week 4: clinging to the bottom of the test tube, partially clinging and partial suspension, complete suspension upon light oscillation, and multi‐site aggregation and clinging. Such an interesting phenomenon leads to speculation that the appearance of suspended matter with unstable morphologies is likely to be an intermediate CNPs form that appears without fully reaching the solid‐phase transformation conditions.

Observing the growth curves, it is evident that CNPs exhibit lag and logarithmic growth phases, with no significant trend toward entering the stationary and decline phases. Growth continues to increase steadily over the culture time. This is consistent with the growth rates mentioned in the literature (Kajander et al., 2003; Lu et al., 2012). The OD values were higher in the SUBDP group compared to the SUPDP group from week 4, suggesting that the SUBDP group may have a higher number of isolated CNPs with greater agglomeration ability.

### 4.2 ALP expression level in CNPs

ALPs is a homodimeric enzyme that was discovered to be one of the first key actors in the osteogenesis process (Zaher et al., 2020). It is thought to promote the mineralization process by hydrolyzing inorganic pyrophosphate (PPi) and raising the concentration of inorganic phosphates (Vimalraj, 2020). To acknowledge, although pathological calcification was thought to be a completely degenerative, uncontrolled process, the mechanism should be similar to physiological calcification (Vidavsky et al., 2021). Some studies have concluded that overexpression of tissue‐nonspecific alkaline phosphatase (TNAP) in endothelium leads to arterial calcification (Savinov et al., 2015), while TNAP inhibition protects against it in mouse models (Romanelli et al., 2017; Tani et al., 2020). Accordingly, ALPs are recognized as involved in the pathogenesis of ectopic calcification (Haarhaus et al., 2022). Considering ALP's roles in biomineralization and pathological calcification, we hypothesize its participation in CNP‐induced mineralization as nucleation cores using environmental components. ALP levels showed a decreasing trend over the culture time with varying degrees of differences between the CNPs‐positive group the culture‐negative group and the control group in both supragingival and subgingival dental plaque samples. These findings lead to the tentative proposal that ALP's involvement in CNP formation might occur once a certain threshold is reached. Several considerations regarding the source of ALP arise:

a) CNPs might inherently secrete ALP during growth, thereby contributing to mineralization. However, the challenge arises from the inability to extract CNPs' nucleic acids, casting uncertainty on their classification as living organisms.
b) ALP derived from FBS. The presence of ALP in the control group suggests its potential occurrence in the FBS in the culture medium. Additionally, the ALP level was the same as before or even slightly increased after fluid replacement (e.g., week 3), in both SUPDP and SUBDP groups. FBS is a main supplement used in cell culture. It encompasses diverse components such as extracellular vesicles, lipoproteins, protein aggregates, and RNA. However, it's crucial to acknowledge that FBS composition can vary among different batches, potentially introducing contaminants like endotoxins, mycoplasma, viruses, and prion proteins, which might influence experimental outcomes (Urzì et al., 2022). Whether the inclusion of CNPs isolates triggers alterations in FBS‐based ALP levels remains unknown.
c) Effects from the microenvironment around the sampling site. Salivary/gingival crevicular fluid (GCF)‐ALP emerges as a biomarker for periodontitis, displaying elevated expression in this pathological context. Fibroblasts, polymorphonuclear leukocytes, and various oral bacteria, particularly Gram‐negative microorganisms in subgingival plaque, contribute to increased ALP levels in response to periodontal tissue damage (Rasaei et al., 2022; Dabra & Singh, 2012). Anatomical regions exposed to elevated ALP concentrations could potentially foster CNP formation. Furthermore, this might elucidate the higher isolation rate of CNPs from subgingival plaque compared to supragingival plaque.

### 4.3 Morphological observation of CNPs

Regarding morphology, the CNPs isolated from plaque were similar to those in previous literature (Fayez Hass et al., 2021; Kajander et al., 2003; Kunishige et al., 2020), which were round and small granular under light microscopy. HE and ARS staining suggested the presence of Ca salt components. TEM revealed round, oval, and ellipsoid particles with a high‐density linear shadow, indicating encasement of the calcified shell. Finer scattered particles between the particles suggest CNPs use inorganic ions in the surrounding environment to form a mineralized shell.

### 4.4 CNP chemical composition analysis

The investigation into CNPs' chemical composition revealed a measured Ca/P ratio of 0.6753, differing from that of both HA and dental calculus 1.67 (Munir et al., 2022). This divergence could stem from a relatively low early‐stage Ca concentration during CNP formation, possibly indicating an incomplete maturation process. Stability was gradually achieved with increased Ca uptake, which was consistent with our macroscopic observation that some samples became semi‐suspended or suspended upon shaking. FTIR analysis showed significant functional groups common to HA and dental calculus, except for the methyl group. According to the protein‐mineral theory, Peng (Kunishige et al., 2020) identified fetuin‐A, albumin, and apolipoprotein A1 as primary protein components within CNPs. The presence of methyl group indicates the presence of organic matter, after ruling out the influence of compounds such as alcohol on the experimental procedure. It is therefore likely that this methyl group is derived from some protein component of the culture process, and its specific contributors need to be further studied.

XRD examination of CNPs' crystal morphology revealed consistency in peak patterns across six samples, indicating the stability of CNPs' primary chemical components—predominantly HA and tricalcium phosphate, mirroring dental calculus's chemical makeup from database comparison. This demonstrates that CNPs produce compounds similar to dental calculus during the culture phase.

### 4.5 Limitations and prospects

In this preliminary study, an in vitro isolation and culture method for CNPs derived from dental plaque was successfully established. Nevertheless, certain limitations persist. CNPs have been characterized only in terms of morphology and chemical composition, without sufficient evidence of the origin of their core components. At present, mineral‐organic nanoparticles are widely accepted in academic circles, with fetuin‐A thought to function as nucleation seeds that expand through ion sedimentation to produce bigger amorphous nanoparticles (Can Demirdöğen, 2019; Martel et al., 2018). Consequently, exploring the levels and types of proteins in their components should be an urgent issue to be addressed due to the failure to validate the nucleic acid sequences. On the other hand, in addition to inducing pathological calcification formation, the cytotoxic effect of CNPs on periodontal tissues and the symbiotic state with periodontal pathogenic bacteria in the periodontal microbial ecosystems represent promising directions for future exploration.

## 5 CONCLUSION

Within the limitations of this present study, we unveiled the occurrence of CNPs in dental plaque of individuals suffering from periodontitis. Notably, there appears to be a proclivity for subgingival plaque to exhibit higher CNP concentrations than supragingival plaque. These cultures exhibit morphological and chemical similarities to dental calculus, and variations in ALP levels may indicate their potential involvement in mineralization processes. The findings suggest a potential correlation between CNPs and periodontal disease, underlining the necessity for further research into their specific mechanisms.

## AUTHOR CONTRIBUTIONS

Conceptualization: Yang L. Data curation: Gu Y. Formal analysis: Bai GH. Funding acquisition: Liu JG. Investigation: Wang Siwei Wang. Methodology: Wang Siwei Wang. Software: Fan Q. Validation: Guan XY. Visualization: Yuan J. Writing ‐ original draft: Wang Siwei Wang. Writing ‐ review & editing: Yuan J, Liu JG.

## CONFLICT OF INTEREST STATEMENT

The authors declare no conflict of interest.

## ETHICS STATEMENT

The present study protocol was reviewed and approved by the ethics committee of the affiliated Stomatological Hospital of Zunyi Medical University (ZYKQ‐IRB‐CT‐2019‐002). Informed consent was submitted by all subjects when they were enrolled.

## Data Availability

The data that support the findings of this study are available on request from the corresponding author.
